# *Trigonella foenum-graecum* L. seed extract modulates biochemical and histomorphological changes in therapeutic model of high-fat diet-fed ovariectomized rats

**DOI:** 10.1007/s13205-023-03707-8

**Published:** 2023-07-28

**Authors:** Takkella Nagamma, Anjaneyulu Konuri, Kumar M. R. Bhat, Padmanabha E. G. Udupa, Yogendra Nayak

**Affiliations:** 1 Department of Biochemistry, Manipal TATA Medical College, Manipal Academy of Higher Education, Manipal, Karnataka 576104 India; 2 Department of Anatomy, Manipal TATA Medical College, Manipal Academy of Higher Education, Manipal, Karnataka 576104 India; 3 Department of Anatomy, Ras Al Khaimah College of Medical Sciences, RAK Medical and Health Science University, Ras Al Khaimah, UAE; 4 Department of Biochemistry, Kasturba Medical College, Manipal Academy of Higher Education, Manipal, Karnataka 576104 India; 5 Department of Pharmacology, Manipal College of Pharmaceutical Sciences, Manipal Academy of Higher Education, Manipal, Karnataka 576104 India

**Keywords:** Ovariectomy, High-fat diet, Leptin, Adiponectin, PPAR-γ, α-smooth muscle actin

## Abstract

This study investigates the therapeutic effect of petroleum ether fraction of *Trigonella foenum-graecum* L. (PE-TFG) seed extract in ovariectomized rats fed with high-fat diet. Rats were randomly grouped into sham ovariectomy (S.OVX), ovariectomy + high-fat diet (OVX + HFD), and treatment groups. The blood samples were collected, and lipid profile, glucose, hepatic markers, and inflammatory markers were estimated. Liver, kidney, and common carotid artery were isolated for histopathological observations. Liver samples were tested for antioxidant, oxidative stress markers, mRNA expression of adiponectin, and PPAR-γ. PE-TFG treatment significantly decreased total cholesterol (18%), LDL (20%), hepatic markers (28%), leptin (17%), TNF-α (21%), and increased mRNA expression of adiponectin and PPAR-γ. There was also micro- and macro-hepatic steatosis, inflammation in the liver, deteriorated tubules in the kidney, and increased tunica intima and media thickness of the common carotid artery. These pathological alterations were reversed with PE-TFG administration. This impact might be linked to phytoestrogens and other components in PE-TFG such as diosgenin, phenols, and flavonoids.

**Supplementary Information:**

The online version contains supplementary material available at 10.1007/s13205-023-03707-8.

## Introduction

Phytoestrogens are plant-derived naturally occurring compounds that exhibit an estrogen-mimic effect in the body and are used as natural substitutes for hormone replacement therapy to ameliorate menopausal symptoms. They are structurally identical to estrogen secreted endogenously in the body and hence, act as estrogen due to their affinity to estrogen receptors in the body (Surguchov et al. 2021; Ionescu et al. 2021). The phytoestrogens and other polyphenols in the seeds of Trigonella foenum-graecum (fenugreek; TFG) modulate the inflammation associated with dyslipidemia and menopause (Visuvanathan et al. 2022). TFG seed powder decreases the synthesis of triglycerides by delaying the absorption of glucose and fatty acids, which are the substrates for triacylglycerol (Geberemeskel et al. 2019). The hypocholesterolemic effect of the TFG is also due to increased fecal excretion of bile acids and the depletion of cholesterol in the liver (Bruce-Keller et al. 2020). TFG also contains saponins, alkaloids, and flavonoids as the primary chemical components responsible for anti-inflammatory activity. TFG seed extract decreases the cytokines IL-6 and TNF-α in diabetic rats (Joshi et al. 2015). Acute and subacute toxicity studies revealed that TFG seed extract has significantly reduced AST, ALT, ALP, LDH, and GGT levels against sodium arsenite-induced toxicity in mice (Biswas et al. 2019). TFG administration also is accredited to its estrogenic constituents, such as trigonelline, diosgenin, and flavonoids (saponins) (Younesy et al. 2014).

Rats are a standard animal model for studying the effects of estrogen deficiency and its metabolic effects. The ovariectomy in rats mimics human menopause (Yousefzadeh et al. 2020). Estrogen deficiency is frequently associated with increased food consumption and body weight. Obesity is an additional concern in menopausal women and may impact oxidative stress in the body’s vital organs, including the liver (Brady 2015). Feeding rats with high-fat diet (HFD) was a helpful model of the effects of dietary fat in humans. HFD is a significant risk factor for various diseases, including metabolic and cardiovascular disease (CVD). HFD long-term results in nonalcoholic fatty liver disease (NAFLD). Inflammatory signals such as TNF-α promote the expression of leptin and leptin receptors (La Cava 2017). Adiponectin is an insulin-sensitizing hormone with anti-inflammatory and anti-atherogenic properties. Adiponectin level was decreased in obese rodents and humans. Increased expression of PPAR-γ protects from the insulin resistance associated with obesity. From our previous studies, we observed that ovariectomy and HFD-induced obesity could be one of the significant causes of reduced adiponectin and PPAR-γ expression (Nagamma et al. 2019b, 2021, 2022). In a previous study, we found that PE-TFG seed extract had anti-inflammatory activity in an ovariectomy model (Nagamma et al. 2022). This study aimed to look into the therapeutic efficacy of a petroleum ether fraction of *Trigonella foenum-graecum* L. seed extract (PE-TFG) in ovariectomized rats fed with HFD.

## Materials and methods

### Materials

The TFG seeds (Pro Natural, 100% Organic Seeds) PE-TFG was prepared as reported earlier (Nagamma et al. 2019a) and later formulated in 0.5% Carboxymethyl cellulose (CMC) and given orally at 50 mg/kg/day. Diosgenin (Sigma-Aldrich, 98% purity) was formulated in 0.5% CMC and given orally at 50 mg/kg/day. Atorvastatin was formulated in 0.5% CMC and given orally at 10 mg/kg/day. 17β-estradiol (E8515-5 G, Sigma-Aldrich) was formulated in sesame oil and injected subcutaneously at 100 μg/kg/day.

High-fat diet composition (1 kg): Pellet powder—325 g, Lard—400 g, Casein—100 g, Sucrose—100 g, Cholesterol—50 g, Vitamin—20 ml, and Choline bitartrate—5 g (Buettner et al. 2007).

### Methods

#### Procurement of Sprague Dawley rats

The animal experiments were carried out as per the national and international guidelines, and the institutional animal ethics committee approval was taken prior to the investigation. The healthy female Sprague Dawley rats weighing 150–200 g and aged 2–3 months were used in this study. Rats are maintained at central animal facilities with utmost care. Ovariectomized rats and the sham ovariectomy rats were fed with a standard pellet diet and water ad libitum, before they were randomized into treatment groups.

#### Ovariectomy for induction of menopause and treatment planner

**Fig. 1:**
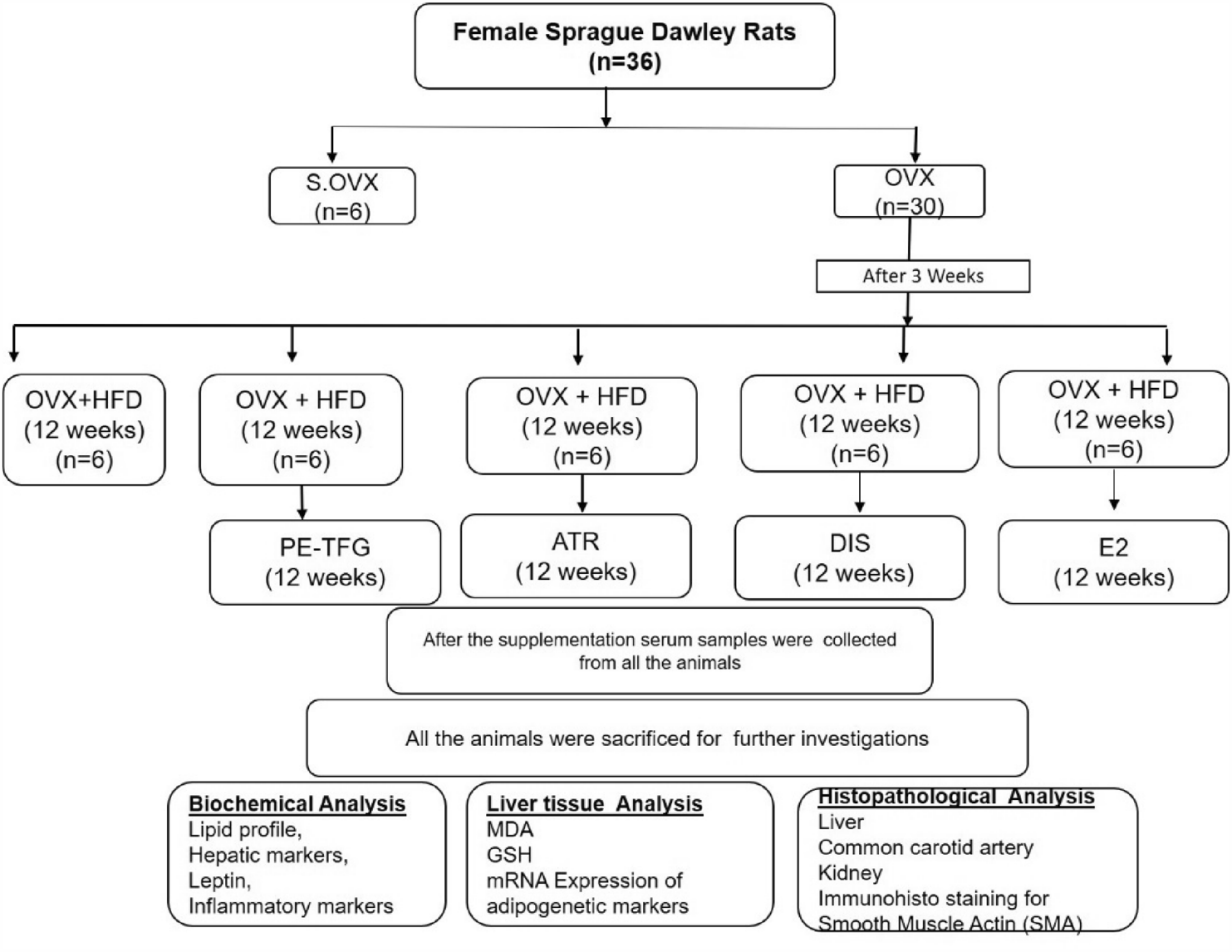
Study design. Six rats in each group (*n* = 6). *S.OVX* sham ovariectomy, *OVX* ovariectomy, *HFD* high-fat diet, *PE-TFG* petroleum ether fraction of *Trigonella foenum-graecum*, *ATR* atorvastatin, *DIS* diosgenin, *E2* 17β-estradiol, *MDA* malondialdehyde, *GSH* glutathione

Thirty-six rats were randomly divided into two groups: Sham ovariectomy (S.OVX) and OVX. Ketamine (50 mg/kg body weight) and xylazine (5 mg/kg body weight) were administered intraperitoneally (i.p.) to the OVX rats to induce sedation. After assessing the withdrawal and blinking reflexes, the rats were prepared for lower abdominal surgery, and bilateral ovariectomy was performed (Anjaneyulu et al. 2018). The above surgical technique was performed in sham-operated rats without removing the ovaries. Rats were grouped as sham ovariectomy (S.OVX), Ovariectomy + High-fat diet (OVX + HFD). Three weeks after ovariectomy, all rats were fed with HFD for 12 weeks. After 12 weeks of HFD, rats were further divided into four groups and treated with PE-TFG seed extract (OVX + HFD + PE-TFG), ATR (OVX + HFD + ATR), DIS (OVX + HFD + DIS), and E2 (OVX + HFD + E2), respectively, for 12 weeks. The study plan was shown in the pictorial form in Fig. 1

Body weight was noted each week, and the BMI (Body mass index) for the rats in all the groups was calculated using the formula: Body weight (g)/Body length cm^2^. A fasting sample (2 ml blood) was taken from each rat after 12 weeks of supplementation by puncturing the retro-orbital plexus, and serum was collected. After all the rats were sacrificed, liver, kidney, and common carotid artery (CCA) were immediately parted for further study. The mRNA expression of adiponectin and PPAR-**γ**, antioxidant, and oxidative stress indicators was measured in the liver tissue. A small portion of the kidney, liver, and CCA was fixed in 10% formalin for histological analysis.

## Biochemical Parameters

### Estimation of serum lipid profile

Serum TC, HDL, and triglycerides (TG) were estimated enzymatically using standard kits obtained from Aspen Ltd. New Delhi, India. Serum LDL was assessed by using the Friedewald formula (Friedewald et al. 1972). Atherogenic index (AI) and Coronary Risk Index (CRI) was calculated as log (TG/HDL) and TC/HDL, respectively (Abedinzade et al. 2015).

### Estimation of glucose

Immediately after collecting the blood samples, a small drop was placed on the test strip of the Accu-check glucometer, and the reading was taken. Results were expressed in mg/dl.

### Hepatic enzymes

Aspartate aminotransferase (AST) and Alanine aminotransferase (ALT) levels were assessed using standard kits obtained from Aspen Ltd., New Delhi, India. The sample was (100 µl) mixed with 1 ml of working solution and then, incubated at 37°C for a minute. The semi-autoanalyzer was used to estimate the enzyme activity, and the absorbance (OD) was fixed at 340 nm as per the instructions given in the kits. The results are expressed as U/l.

### Estimation of the thiobarbituric acid reactive substance

A solution of 100 μl of homogeneous tissue was taken in a clean test tube, and 1000 μl of 0.67% thiobarbituric acid and 500 μl of 20% trichloroacetic acid were added. Test tubes were then incubated at 100ºC for 20 min in a water bath. Subsequently, the test tubes were cooled, and the supernatant was transferred into Eppendorf vials. The vials were then centrifuged at 12,000 rpm for 5 min. Supernatant absorbance was read at 532 nm 1995). The results were expressed as nmol/mg of wet tissue (Nagamma et al. 2019b).

### Estimation of reduced glutathione

A sample of 100 µl of liver homogenate was added to 100 µl of 5% trichloroacetic acid (TCA) solution and centrifuged for 5 min at 5000 rpm. 25 µl of supernatant was added to 150 µl of sodium phosphate buffer (PBS 0.2 M, pH 8.0) and 25 µl of 0.6 mM 5,5–dithio-bis-(2-nitrobenzoic acid) (DTNB) in 96-wells of the micro-well plate. This plate was then incubated at room temperature for 10 min, and absorbance was read at 412 nm by using an ELISA reader (Nagamma et al. 2019b).

### Serum leptin estimation

Serum leptin was assessed by using RayBio Leptin ELISA kit. The ELISA kit was a solid-phase enzyme immunoassay based on the sandwich principle for the quantitative in vitro diagnostic measurement of leptin in serum. The color intensity is directly proportional to leptin concentration. The values were expressed as pg /ml.

### Estimation of tumor necrosis factor-alpha (TNF-$\alpha$)

Serum TNF**-**$\alpha$ levels were quantified by ELISA method as per the protocol mentioned in the ELISA kit (Catalog no: ELR-TNFa-CL, RayBio^®^). The concentration was calculated by comparing OD of the samples to the standard curve. The values were expressed as pg/ml.

### Expression of adiponectin and PPAR-γ

The RNA was obtained from frozen liver tissue using PureLink RNA isolation kit (Invitrogen, USA), and cDNA was prepared by using Superscipt III First-strand synthesis kit (Invitrogen, USA) followed by manufacturer instructions. Only the cDNA template was amplified in all primer sets targeting the following genes, GAPDH, PPAR-γ and adiponectin. PCR was carried out in 25 µl reaction mixture having 1X PCR buffer (Thermo scientific, USA), 1 µg of forward and reverse primers (Bioserve, India), 0.25 mM dNTP mix (Applied Biosystems, USA) and 2-units of DNA polymerase (Thermo Scientific, USA) in PeqSTAR Thermal cycler (PeqLab, Germany). PCR products were observed in agarose gel (2%) stained with ethidium bromide and snapped on a UV light transilluminator. The sequence of the primers is as follows: GAPDH Forward –CTAGAGACAGCCGCATC; GAPDH Reverse- GGGTAGAGTCATACTGGAAC: Adiponectin Forward-AATCCTGCCCAGTCATGAAG; Adiponectin Reverse- CATCTCCTGGGTCACCCTTA: PPAR-γ Forward- CATGACCAGGGAGTTCCTCAA. PPAR-γ Reverse- GCAAACTCAAACTTAGGCTCCATAA. ImageJ software was used to obtain the intensity of the bands and represented as a graph (Nagamma et al. 2022).

### Histopathological analysis

Formalin-fixed liver, kidney, and common carotid artery tissue were processed and stained with hematoxylin and eosin (H&E). De-paraffinization was performed with two changes of xylol for 5 min each. Then, slides were hydrated with descending grades of alcohol (100%, 90%, 70%, and 50%) for 2 min and 5 min with distilled water. Staining procedure: sections were stained with hematoxylin for 5 min, then kept under running tap water for 10 min for bluing. After bluing, it was stained with 1% aqueous eosin for 1 min. Dehydrated with 90 and 100% alcohol, cleared with xylene, and then, mounted with DPX (Xu et al. 2013). Light microscopy was used to compare the histological changes between the groups by expert anatomist at the Department of Anatomy, Kasturba Medical College, Manipal. The reports were then compiled for comparison.

### Statistical analysis

Graph Pad Prism Version 5.0 used one-way ANOVA with Bonferroni’s post hoc test to evaluate the data. The results were presented as mean ± SEM. *p* ≤ 0.05 was considered significant.

## Results

### Effect of PE-TFG on bodyweight and BMI

**Fig. 2:**
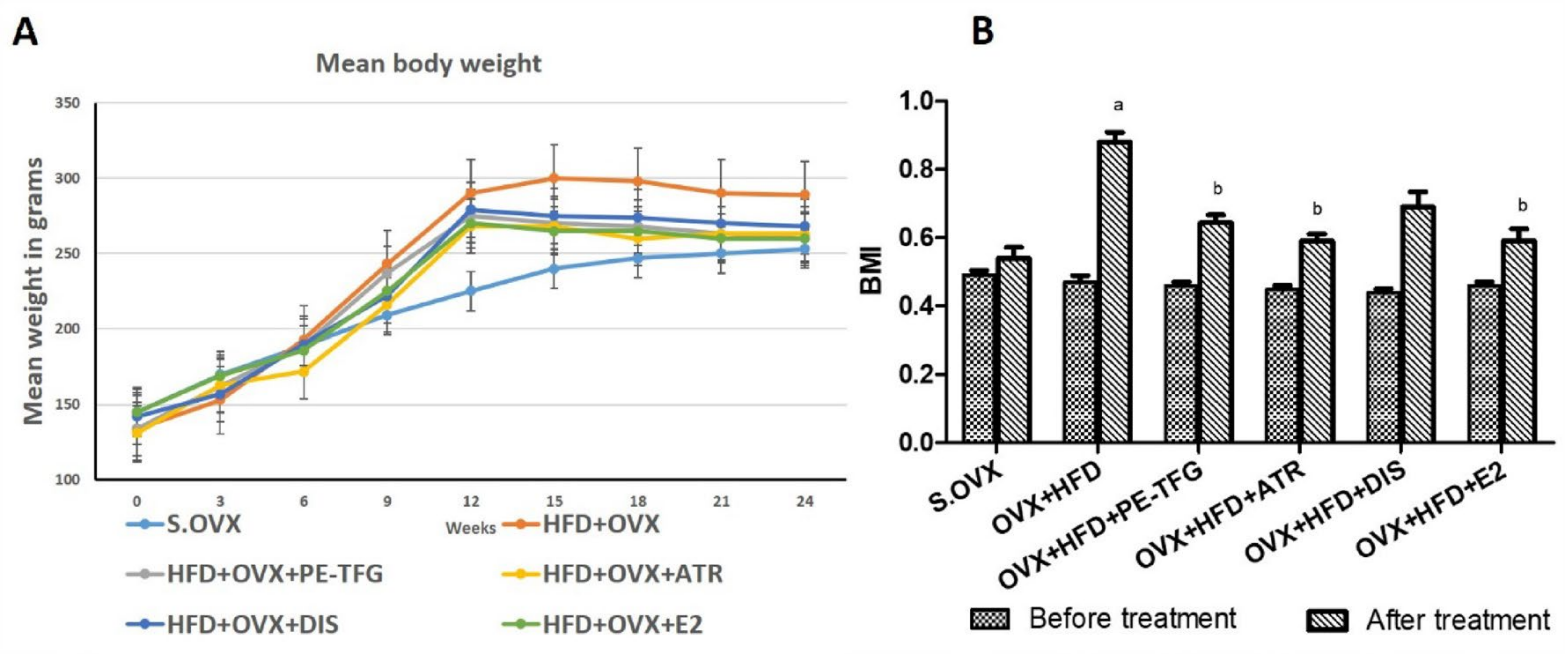
Body weight and BMI in the OVX group fed HFD. **A** Mean body weight; **B** BMI. The values are expressed in mean ± SEM. S.OVX vs. OVX + HFD: ^a^*P* < 0.001; OVX + HFD vs. OVX + HFD-treated groups ^b^*P* < 0.05 (One-way ANOVA, Bonferroni’s test). *S.OVX* sham ovariectomy, *OVX* ovariectomy, *HFD* high-fat diet, *PE-TFG* petroleum ether fraction of *Trigonella foenum-graecum*, *ATR* atorvastatin, *DIS* diosgenin, *E2* 17β-estradiol

The body weights of the rats were monitored for 24 weeks at weekly intervals, and the results are represented in Fig. 2A. All the groups were fed with HFD for 12 weeks. There was no substantial difference in mean body weight between groups at zero weeks. At the end of 24th week, body weight increased significantly (*P* < 0.001) in OVX + HFD group compared with S.OVX group. There was a significant decrease (*P* < 0.01) in body weight among the PE-TFG, ATR, and E2 treated groups.

At the beginning of the experiment, there was no significant difference in BMI between the groups (Fig. 2B). However, there was a significant increase (*P* < 0.001) in BMI in the OVX + HFD group after 24 weeks compared to S.OVX. PE-TFG, ATR, DIS, and E2 treatment suggestively decreased (*P* = 0.02) BMI levels compared to the OVX + HFD group.

## Biochemical test results

### Effect of PE-TFG on lipids, glucose, AST, and ALT

The results are represented in Table 1. TC, TG and LDL, glucose, AST, and ALT were significantly increased (*P* = 0.001) in the OVX + HFD group compared to S.OVX. However, after treatment with test material lipid profile, glucose, AST, and ATL levels were significantly decreased (*P* < 0.05). HDL was elevated but not to a significant level. The percentage of reduced TC (18%), TG (22%), LDL (20%), glucose (19%), AST (28%), and ALT (18.5%) after treatment with PE-TFG.

**Table 1:** The effect of PE-TFG on the blood lipid profile, glucose, AST, and ALT

Parameters	S.OVX	OVX + HFD	OVX + HFD + PE-TFG	OVX + HFD + ATR	OVX + HFD + DIS	OVX + HFD + E2
Total Cholesterol (TC, mg/dl)	73.9 ± 3.8	120 ± 3^a^	97.7 ± 3.4^b^	101 ± 2.6^b^	104 ± 4.8^b^	100 ± 2.6^b^
Triglycerides (mg/dl)	92.1 ± 5.4	172 ± 7.1^a^	134 ± 8.8^b^	130 ± 5.6^b^	138 ± 6.6^b^	133 ± 5^b^
LDL (mg/dl)	37 ± 4.7	60.4 ± 8.3^a^	48 ± 6.5^b^	50 ± 6.7	52 ± 7.1	49.9 ± 6.6
HDL (mg/dl)	24 ± 1.6	16 ± 0.6^a^	19 ± 0.8	20 ± 0.9	18 ± 0.9	20.5 ± 0.7
Atherogenic index (AI)	0.57 ± 0.05	1 ± 0.02^a^	0.82 ± 0.03^b^	0.76 ± 0.02^b^	0.85 ± 0.01	0.77 ± 0.03^b^
Coronary risk index (CRI)	3 ± 0.29	7.4 ± 0.4^a^	5.2 ± 0.3^b^	5 ± 0.26^b^	5.6 ± 0.3^b^	4.9 ± 0.2^b^
Glucose (mg/dl)	77 ± 4	136 ± 1.2^a^	109 ± 1.9^b^	101 ± 1.1^b^	98 ± 1.3^b^	100 ± 1.6^b^
AST (U/L)	26.5 ± 2.3	73 ± 2.4^a^	52.8 ± 2.4^b^	58.2 ± 7.1	66.6 ± 3.6	56.1 ± 4.2
ALT (U/L)	36.3 ± 3	60 ± 5^a^	49.1 ± 2^b^	49.3 ± 3.2^b^	53.5 ± 4.1	51.6 ± 2.6

The values are stated in mean ± SEM. S.OVX vs. OVX + HFD: ^a^*P* = 0.001; OVX + HFD vs. OVX + HFD treated groups: ^b^*P* < 0.05 (One-way ANOVA, Bonferroni’s test)

*S.OVX* sham ovariectomy, *OVX* ovariectomy, *HFD* high-fat diet, *PE-TFG* petroleum ether fraction of *Trigonella foenum-graecum*, *ATR* atorvastatin, *DIS* diosgenin, *E2* 17β-estradiol

### Effect of PE-TFG on TBARS and GSH in liver

**Fig. 3:**
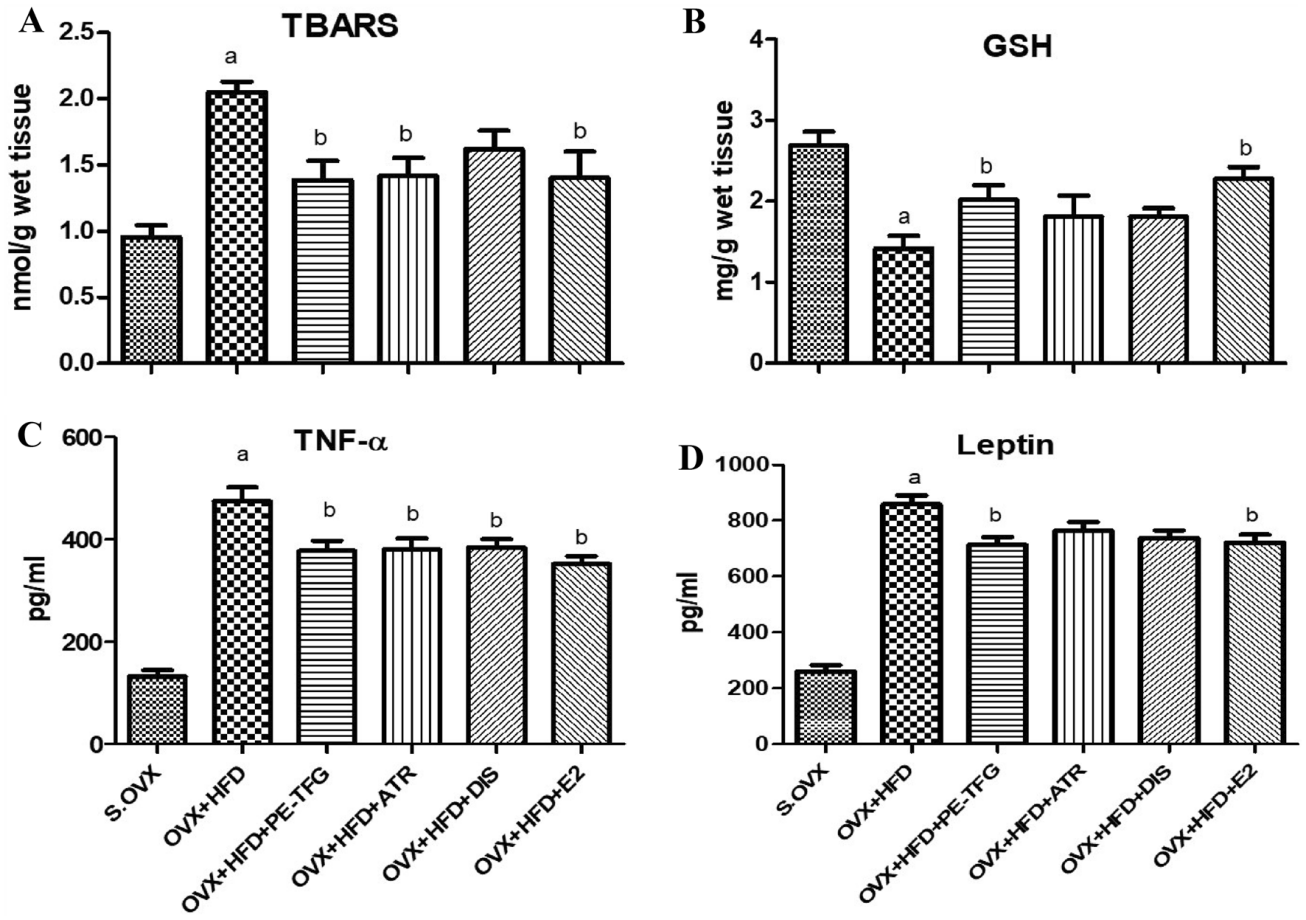
Liver lysate and serum parameters in OVX-HFD rats. **A** TBARS; **B** GSH; **C** Serum TNF-alpha; **D** Serum leptin. S.OVX vs. OVX + HFD: ^a^*P* = 0.01; OVX + HFD vs. OVX + HFD-treated groups: ^b^*P* < 0.05. TBARS: thiobarbituric acid reactive substances; GSH: reduced glutathione; TNF-α: tumor necrosis factor; Leptin. *S.OVX* sham ovariectomy, *OVX* ovariectomy, *HFD* High-fat diet, *PE-TFG* petroleum ether fraction of *Trigonella foenumgraecum*, *ATR* atorvastatin, *DIS* diosgenin, *E2* 17β-estradiol

TBARS level in the liver of OVX + HFD (2 ± 0.08) was significantly elevated (*P* = 0.01) compared to S.OVX. PE-TFG seed extract, ATR, DIS, and E2 significantly decreased (*P* = 0.02), the TBARS levels compared to the OVX + HFD group. The percentage of TBARS levels reduced by PE-TFG (35%), ATR (30%), and E2 (30%) compared to the OVX + HFD GSH level in the liver decreased (*P* = 0.002) in OVX + HFD compared with S.OVX. After treatment with PE-TFG seed extract (42.8%) and E2 (57%), GSH levels significantly increased (*P* < 0.05) (Fig. 3A and B).

### Effect of PE-TGF on tumor necrosis factor-α (TNF-α) and leptin in serum

TNF-α was reduced by 21%, 20%, 19%, and 26% after being treated with PE-TFG, DIS, ATR, and E2, respectively, compared to the OVX + HFD group. Leptins significantly increased (*P* = 0.001) in the serum of the OVX + HFD group compared to the S.OVX group. PE-TFG seed extract decreased leptin by 17% (*P* < 0.05) and E2 by 16% (*P* < 0.05) compared to the OVX + HFD group (Fig. 3C and B).

### The effects of PE-TGF on the expression of PPAR-γ and adiponectin mRNA in liver

**Fig. 4:**
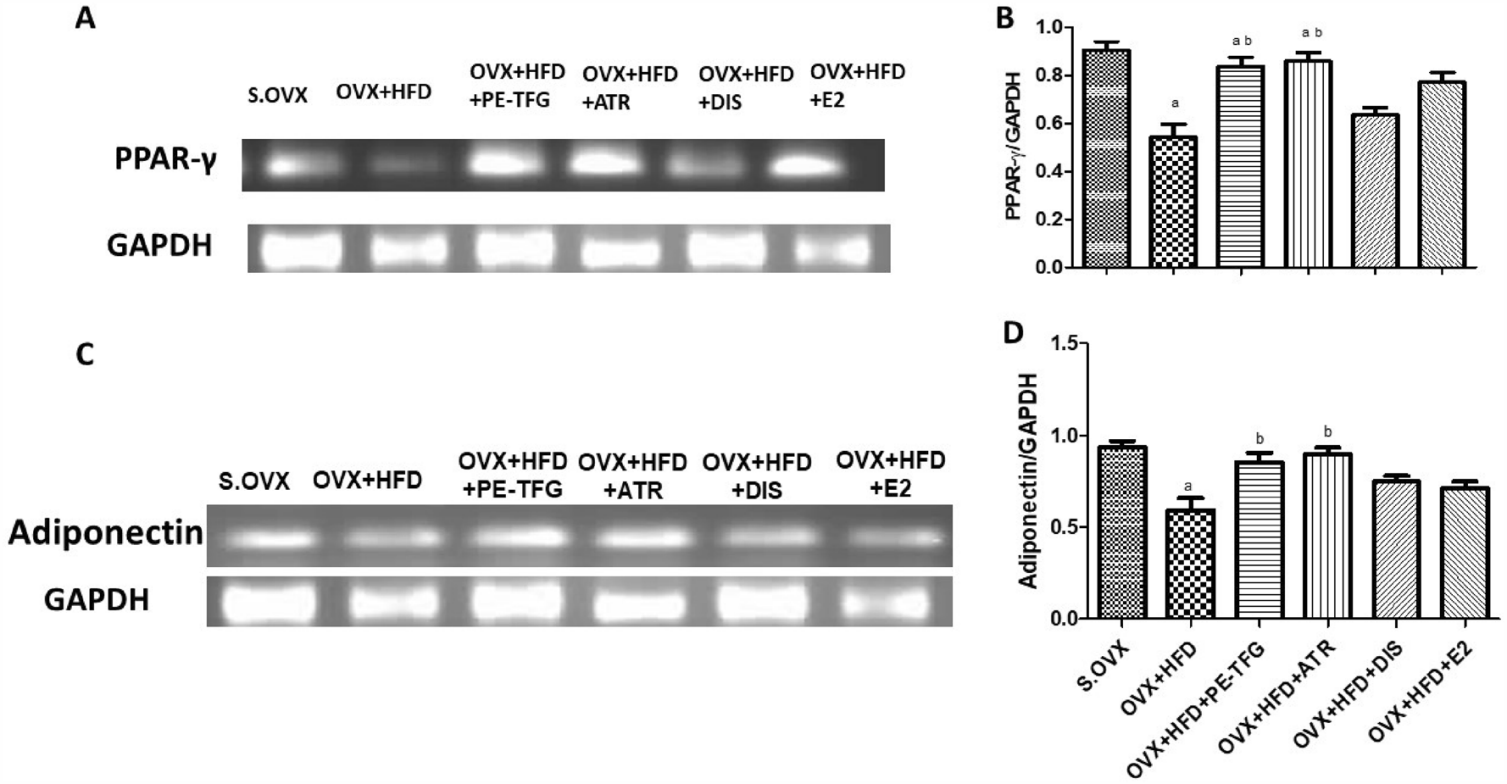
Expression of PPAR-γ, adiponectin, and GAPDH mRNA in rat liver of OVX-HFD therapeutic model. **A** PPAR-γ and GAPDH mRNA expression; **B** Quantification of PPAR-γ; **C** Adiponectin and GAPDH mRNA expression; **D** Quantification of adiponectin expression. S.OVX vs. OVX + HFD: ^a^*P* = 0.01; OVX + HFD vs. OVX + HFD-treated groups: ^b^*P* < 0.05. *PPAR-γ* peroxisome proliferator-activated receptor-gamma, *S.OVX* sham ovariectomy, *OVX* ovariectomy, *HFD* high-fat diet, *PE-TFG* petroleum ether fraction of *Trigonella foenum-graecum*, *ATR* atorvastatin, *DIS* diosgenin, *E2* 17β-estradiol

PPAR-γ expression significantly decreased in OVX + HFD group compared to S.OVX. After treatment with PE-TFG and ATR, PPAR-γ mRNA expression increased significantly compared to OVX + HFD (Fig. 4A).

The adiponectin expression significantly decreased in the OVX + HFD group compared to S.OVX group. The adiponectin mRNA expression significantly increased (*P* < 0.01) after the treatment with PE-TFG and ATR (Fig. 4B).

### Effect of PE-TGF on histopathology

#### Histology of liver

**Fig. 5:**
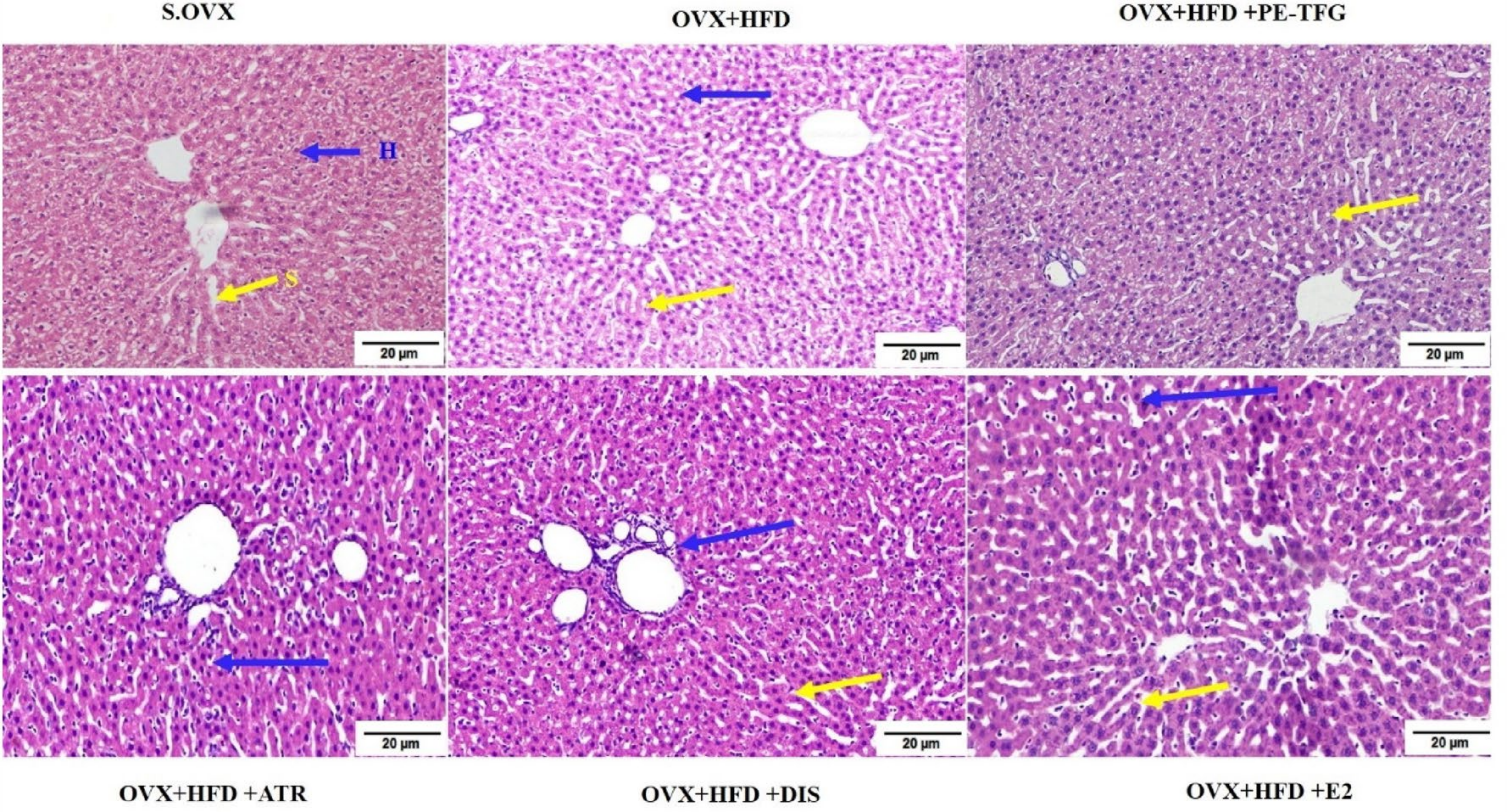
H&E stain of liver in a therapeutic model of OVX group fed with HFD (objective lens, × 10). Yellow Arrow—Sinusoids (S), Blue Arrow—Hepatocytes (H). *S.OVX* sham ovariectomy, *OVX* ovariectomy, *HFD* high-fat diet, *PE-TFG* petroleum ether fraction of *Trigonella foenum-graecum*, *ATR* atorvastatin, *DIS* diosgenin, *E2* 17β-estradiol

S.OVX group exhibited standard liver architecture with distinct hepatocytes (H), sinusoidal spaces (S), and well-preserved cytoplasm with prominent nuclei. OVX + HFD group showed dilated sinusoids, inflammation (mild), infiltrations around portal trait, macro- and micro-steatosis. Micro-steatosis and mild inflammation were observed in OVX + HFD + PE-TFG-treated group. Dilation of sinusoid micro-steatosis infiltrations around portal triad was observed in atorvastatin, diosgenin and estradiol-treated groups (Fig. 5).

#### Histology of kidney

**Fig. 6:**
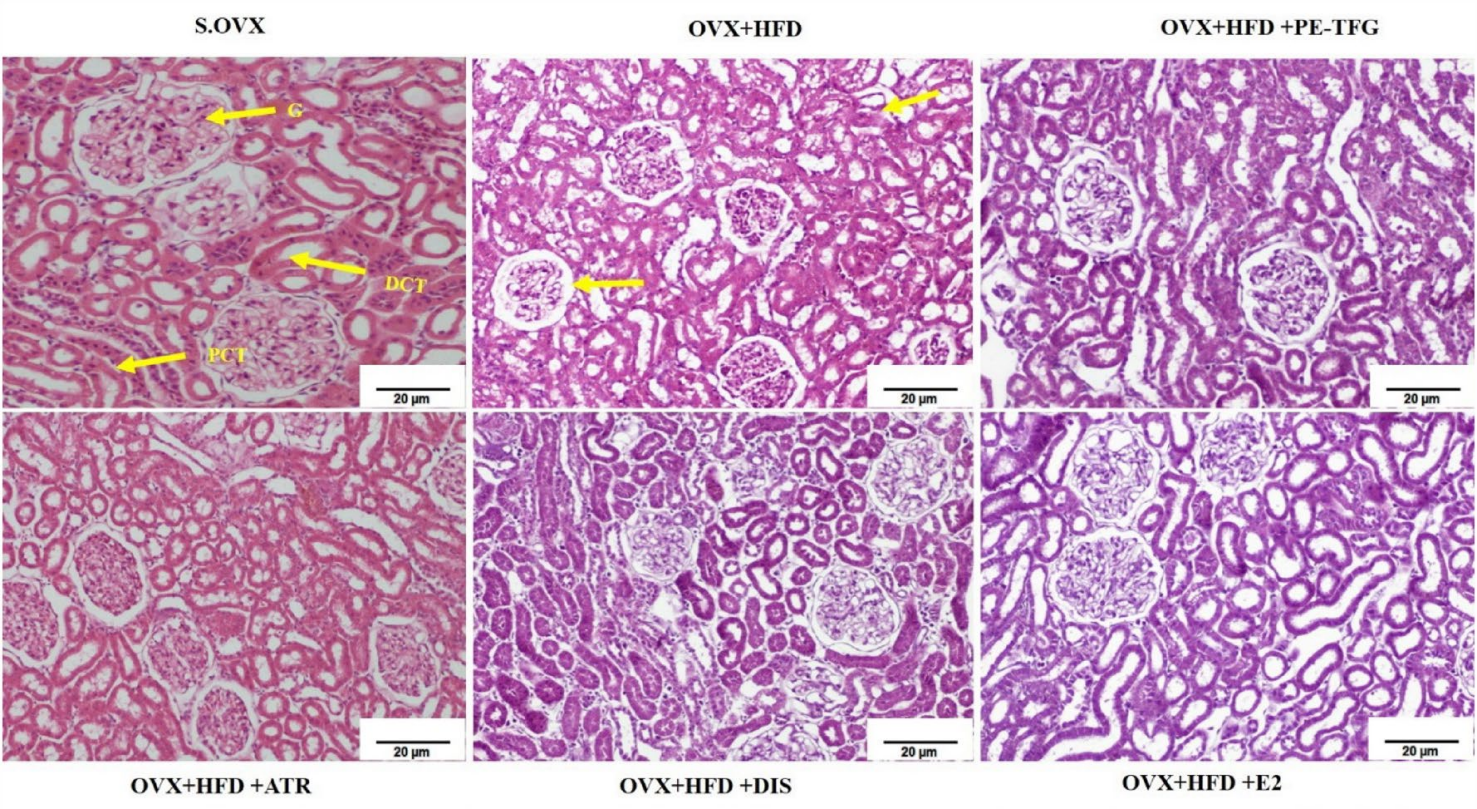
H&E staining of kidney in the therapeutic model of OVX group fed with high-fat diet (objective lens, × 10). *G* glomerulus, *DCT* distal convoluted tubule, *PCT* proximal convoluted tubule. *S.OVX* sham ovariectomy, *OVX* ovariectomy, *HFD* high-fat diet, *PE-TFG* petroleum ether fraction of *Trigonella foenum-graecum*, *ATR* atorvastatin, *DIS* diosgenin, *E2* 17β-estradiol

Normal medullary morphology was observed in S.OVX group. OVX + HFD group presented with glomerular hypertrophy and tubular degeneration. The tubules were regenerated in rats treated with TFG, ATR, DIS, and E2 groups (Fig. 6).

#### Histology of common carotid artery

**Fig. 7:**
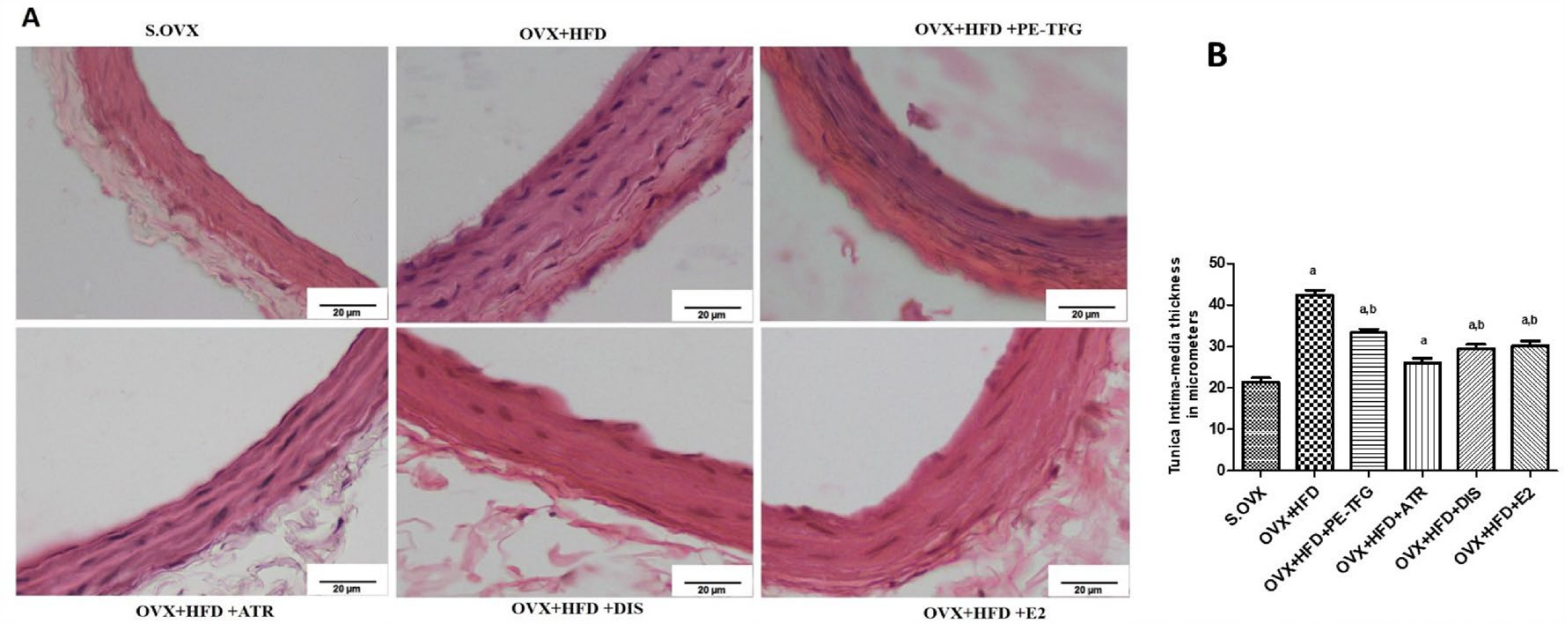
Histopathology of Common Carotid Artery in the therapeutic model of OVX group fed with high-fat diet (objective lens, × 10). **A** H&E staining of Common Carotid Artery; **B** Thickness of tunica intima and media of Common Carotid Artery in a therapeutic model of OVX group fed with high-fat diet. S.OVX vs. OVX + HFD: ^a^*P* = 0.01; OVX + HFD vs. OVX + HFD and treated groups: ^b^*P* < 0.05. *S.OVX* sham ovariectomy, *OVX* ovariectomy, *HFD* high-fat diet, *PE-TFG* petroleum ether fraction of *Trigonella foenum-graecum*, *ATR* atorvastatin, *DIS* diosgenin, *E2* 17β-estradiol

The thickness of tunica intima and media was increased significantly (*P* < 0.001) in OVX + HFD group (42.3 ± 1.2) compared to S.OVX group (22.2 ± 1). The treatment with PE-TFG (33 ± 0.9), ATR (27 ± 1.2), DIS (29 ± 0.9), and E2 (30.3 ± 1.1), the thickness of the artery reduced (Fig. 7).

## Discussion

Our studies have examined PE-TFG supplementation in the therapeutic model of high-fat-induced menopause in rats for its beneficial effects on body weight, biochemical, and other histopathological parameters. Body mass index and fat mass were increased after ovariectomy with HFD. This impact might be attributed to the lack of ovarian hormones, and HFD-induced obesity enhanced the production of proinflammatory cytokines. Following our results, eight weeks of HFD consumption did not significantly alter body weight. A combination of HFD and ovariectomy, on the other hand, raised body weight and gonadal weight (Gorres et al. 2011).

Ovariectomy and HFD are associated with hyperlipidemia, hyperglycemia, and increased AST and ALT levels. These levels were decreased in the range of 18–28% treated with PE-TFG extract. Many studies have reported the hypercholesteremic and hypoglycemic activity of TFG. This effect is mainly attributed to the presence of flavonoids, dietary fibers, which restrict the absorption of lipids from the small intestine, and 4-hydroxy isoleucine which enhances LDL uptake by increasing the activity of lecithin cholesterol acyl transferase and by upregulation of LDL receptor gene expression and also the phytoestrogen content, a large amount of fiber galactose and mannose in the extract (Doyle et al. 2009; Kassaee et al. 2021). Estrogen reduces TC levels by increasing the expression of the hepatic LDL receptor. This is reflected in our rat model, where 17-estradiol-treated OVX-rats significantly reduced serum TC and TG compared to control rats. Our findings are consistent with those of Praveen Kumar et al., who found that supplementing with 0.5 and 1 g/kg/day of FG seed aqueous extract for four weeks reduced TC, TG, and LDL levels while increasing HDL levels (Kumar et al. 2014). Glucose levels were decreased in the PE-TFG-treated groups. These results are supported by Al-Chalabi et al., who stated that diabetic rats were treated with 300 mg/kg of methanolic fenugreek seed extract for 4 weeks, significantly decreasing blood glucose levels (Al-Chalabi et al. 2019). 4-hydroxy isoleucine from TFG seed extract increased insulin secretion in human and rat pancreas and inhibited hepatic glucose production (Zafar and Gao 2016). Pectin, present in the TFG, delays glucose absorption from the intestine. Our study illustrated a significant increase in the AST and ALT levels in OVX rats fed with HFD. The results indicate that feeding with high-fat results in hepatic disorders due to the accumulation of lipids in the liver. The TFG seed extract reduced AST and ALT levels by decreasing the lipid accumulation in the liver. In these results supported by Kandhare et al., rats were administered 20, and 40 mg/kg of TFG exhibited a significant decrease in the serum AST and ALT levels (Kandhare et al. 2017). Estrogen functions as an antioxidant by acting as an electron donor, a free-radical scavenger, and inhibiting lipid peroxidation. Estrogen increases the activity of GSH peroxidase and manganese superoxide dismutase in females. Ovariectomy induces metabolic disruptions, which affect the liver (fatty liver) by generating reactive oxygen species (Kankofer et al. 2007), reducing 35% of the TBARS after treatment with TFG seed extract compared to OVX + HFD. Flavonoids and polyphenols found in TFG seed extract have antioxidant effects. Reduced lipid peroxidation following PE-TFG treatment might potentially be related to saponins-like diosgenin and trigonelline, as well as other flavonoids, which are estrogenic (Belaïd-Nouira et al. 2012; Forni et al. 2019). High-fat diet for the short period influences cholesterol metabolism. Adipose tissue secretes the cytokines like leptin, TNF-α and adiponectin. The results of our research showed that ovariectomy elevated the level of the proinflammatory cytokine TNF-α. In an inflammatory state, TNF-α plays a vital role in developing induced nitric oxide synthase activity, impacts vascular cells, and increases oxidative stress, most of which affect vascular function. PE-TFG decreased the 21% of TNF-α in our Study. According to research, PE-TFG extract significantly reduced serum proinflammatory cytokines IL1,6 and TNF-α (Abedinzade et al. 2015). Diosgenin prevents atherosclerosis by downregulation of TNF-α and NF-κBp65 markers (Binesh et al. 2018). Leptin is crucial for controlling body weight homeostasis, food intake, energy expenditure, and cardiovascular and reproductive function. Estrogen has been shown to influence the tissue-specific expression of leptin receptor-LEPR V2 and leptin in ovariectomized rats (postmenopausal model). Ovariectomy and high-fat diet are associated with increased serum leptin levels by upregulation of body weight and fat mass (Yin et al. 2017). E2 treatment downregulates serum leptin levels, body weight, and fat mass. TFG seed extract contains phytoestrogen that might help in the down-regulation of leptin levels.

Adipocyte size and adipocyte inflammation were reduced by diosgenin present in PE-TFG. PPARγ plays a key role in inflammation, adipogenesis, and cell differentiation. PPARγ activity is decreased in conditions like obesity. The therapeutic action of TFG seed extract may be attributed to an effect similar to insulin, an increase in adiponectin, and the expression of PPARγ protein. Polyphenols present in PE-TFG extract improve the expression of adiponectin; thereby, PPARγ helps in the prevention of hepatic steatosis. TFG extract supplemented for two weeks significantly increased mRNA PPARγ expression of high fructose diet-fed rats for eight weeks (Mohammadi et al. 2016). Mice fed with sanyaku and diosgenin in high-fat diet, lipoprotein lipase and PPAR-γ expression was upregulated in the liver (Hashidume et al. 2018). In support of our study, HFD-fed mice supplemented with 2 g/kg daily TFG increased adiponectin expression in subcutaneous inguinal adipose tissue (Knott et al. 2017). Polyphenols, sapogenins, 4-hydroxy-isoleucine, diosgenin, trigonelline, and alkaloids are abundant in TFG seeds, which contribute to the lowering of proinflammatory cytokine levels (Geberemeskel et al. 2019).

Estrogen deficiency in postmenopausal women affects the dysregulated lipid metabolism, redistribution of fat, and visceral fat accumulation (Ko and Kim 2020). Streptozotocin (120 mg kg) and a high-fat diet for two weeks produced type II diabetes in mice, resulting in increased adiposity and moderate microvesicular steatosis. The liver exhibited typical architecture (no steatosis, inflammation, or necrosis) after supplementation with PE-TFG seed extract (100 mg/kg/BW) (Al-Dabbagh et al. 2018). Conflicting to our study, TFG seed extract at the dose of 100 mg/kg in the streptozotocin-induced diabetic model showed degenerating hepatocytes with shrunken pyknotic nuclei and loss of cellular details in the liver and oral extract showed degenerating hepatocytes and missing cellular details (Baset et al. 2020). Microarchitecture in renal portions of HFD-fed rats revealed dilation of glomerular capillaries as well as other blood vessels, mononuclear cell infiltration in the renal cortex, nephrotic degeneration, including glomerulosclerosis and tubular defects (Salim et al. 2018). TFG protects against morphological injuries of diabetic rat kidneys by increasing the activity of antioxidants and preventing oxidized DNA aggregation (Xue et al. 2011).

Atherosclerosis can be predicted by an increase in the tunica intima/tunica media ratio (TI ⁄TM). Altered TI/TM ratio after ovariectomy, and these results could be signs of early atherosclerosis. TFG seed extract and diosgenin displayed anti-inflammatory effects. Diosgenin prevents the intracellular adhesion molecule (ICAM-1), vascular cell adhesion molecule (VCAM-1), and protein expression involved in the development of atherosclerosis. Diosgenin and TFG are both antioxidants and free radical scavengers (Al-Dabbagh et al. 2018) and prevent H_2_O_2_-induced apoptosis in human endothelium cells. This beneficial impact might be attributed to the metabolites such as flavonoids, polyphenols, and polysaccharides have been shown to be potent free radical scavengers, lipid peroxidation inhibitors, and inhibitors of hepatic stellate cell activation, which disrupt signal transmission and protein cell activation (Srivastava and Srivastava 2018).

## Conclusion

Our study demonstrated the therapeutic benefits of TFG in animal model of menopause. TFG modulated inflammatory changes, increased HDL, showed in vivo antioxidant activity, and increased expression of PPAR-γ, and adiponectin. Additionally, protected the cellular architecture of the liver, kidney, and common carotid artery in ovariectomized and high-fat dietfed rat models. These effects can be attributed to the phytoestrogenic properties of diosgenin, phenols, and flavonoids in TFG seed extract.

## Supplementary Information

Below is the link to the electronic supplementary material.

Supplementary file1 (PPTX 690 KB)

## Data Availability

Most of the data generated in this work is included in the manuscript and the supplementary files. The raw data generated can be shared upon request from the corresponding author.
