# Supplementary material for: Trigonella foenum-graecum L. seed extract modulates biochemical and histomorphological changes in therapeutic model of high-fat diet-fed ovariectomized rats
Source: 3 Biotech. 2023 Jul 28;13(8):285. doi: 10.1007/s13205-023-03707-8 (PMC10382425; doi:10.1007/s13205-023-03707-8)
Supplement: Supplementary file 1 — Supplementary file1 (PPTX 690 KB) [file 13205_2023_3707_MOESM1_ESM.pptx]

## Slide 1
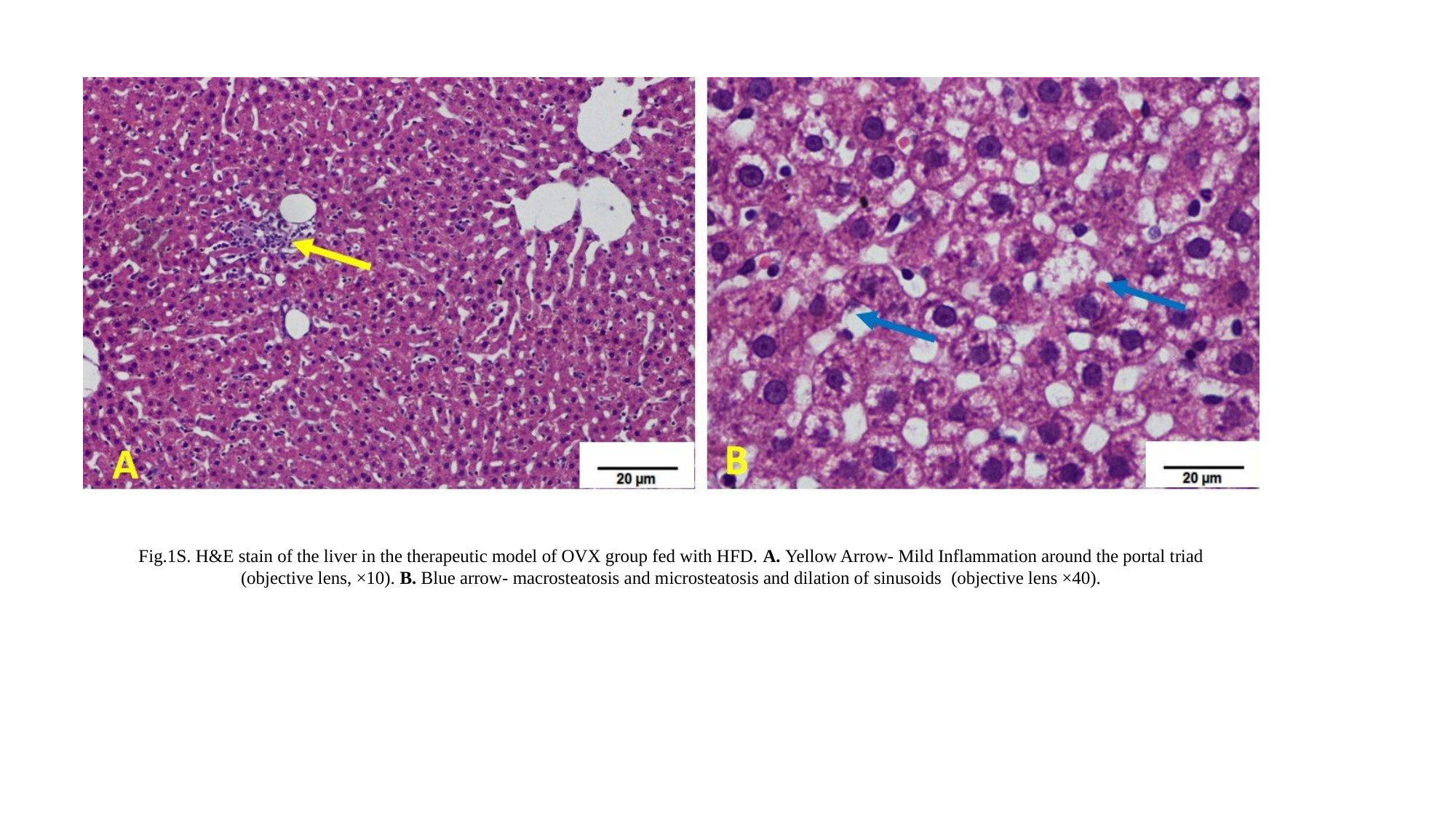

Fig.1S. H&E stain of the liver in the therapeutic model of OVX group fed with HFD. A. Yellow Arrow- Mild Inflammation around the portal triad
 (objective lens, ×10). B. Blue arrow- macrosteatosis and microsteatosis and dilation of sinusoids (objective lens ×40).
